# An Overview on Graphene-Metal Oxide Semiconductor Nanocomposite: A Promising Platform for Visible Light Photocatalytic Activity for the Treatment of Various Pollutants in Aqueous Medium

**DOI:** 10.3390/molecules25225380

**Published:** 2020-11-17

**Authors:** Soumen Mandal, Srinivas Mallapur, Madhusudana Reddy, Jitendra Kumar Singh, Dong-Eun Lee, Taejoon Park

**Affiliations:** 1 Intelligent Construction Automation Center, Kyungpook National University, 80 Daehak-ro, Buk-gu, Daegu 41566, Korea; sou.chm@gmail.com; 2 Department of Chemistry, REVA University, Kattigenahalli, Yelahanka, Bangalore 560024, Karnataka, India; seenuseenum@gmail.com (S.M.); madhusudana.mb@reva.edu.in (M.R.); 3 Innovative Durable Building and Infrastructure Research Center, Department of Architectural Engineering, Hanyang University, 1271 Sa3-dong, Sangnok-gu, Ansan 15588, Korea; jk200386@hanyang.ac.kr; 4 School of Architecture, Civil, Environment, and Energy, Kyungpook National University, 1370, Sangyegk-Dong, Buk-Gu, Daegu 702701, Korea; 5 Department of Robotics Engineering, Hanyang University, 55 Hanyangdaehak-ro, Ansan, Gyeonggi-do 15588, Korea

**Keywords:** graphene oxide, metal oxides, semiconductor, photocatalysis, dyes

## Abstract

Graphene is one of the most favorite materials for materials science research owing to its distinctive chemical and physical properties, such as superior conductivity, extremely larger specific surface area, and good mechanical/chemical stability with the flexible monolayer structure. Graphene is considered as a supreme matrix and electron arbitrator of semiconductor nanoparticles for environmental pollution remediation. The present review looks at the recent progress on the graphene-based metal oxide and ternary composites for photocatalysis application, especially for the application of the environmental remediation. The challenges and perspectives of emerging graphene-based metal oxide nanocomposites for photocatalysis are also discussed.

## 1. Introduction

Graphene is a 2-D material composed of layers of carbon atoms crammed into a honeycomb network and has become an escalating star on the prospect of materials science in the past many years [1,2,3]. Graphene can be used to produce 0-D fullerene, 1-D and 3-D graphitic carbon nanotubes that had been intensively studied for the last ten years [4,5]. Graphene exhibits enthralling assets such as extraordinary conductivity, maximum surface-area-to-volume ratio, a fluorescence-quenching competence by electron or energy-allocation, a quantum Hall effect at room temperature, a bipolar electric field effect laterally with the surface conduction of charge carriers and a tunable band gap [6,7]. Narrow band gap metal oxides are of great interest, due to their efficient utilization of solar energy which signifies an auspicious technology to resolve the global energy and eco-friendly challenges [5,8,9,10]. Furthermore, graphene sheets decorated with metal oxide nanoparticles exhibit the outstanding properties because of the synergetic effect between them [11].

The growth of graphene-based composites provides a significant milestone to multiply the application enactment of metal oxide nanomaterials in photocatalysis, as the hybrids have adaptable and suitable properties with superior performances over the individual oxide nanomaterials. With keeping this in mind, considerable efforts have been made on decorating graphene with metal oxides [12]. Graphene-based materials have also been used as the catalyst in the reactions pertaining to environmental remediation [13]. Graphene oxide-based (GO-based)/reduced graphene oxide-based (rGO-based) materials are used as photocatalysts for pollutant abatement [14,15,16,17].

In this context, focusing on the recent developments, an attempt is made in the present review to discuss the advantages and disadvantages of the composite in comparison with pristine graphene.

## 2. Photocatalysis

Different metal oxides-GO/rGO composites, their photocatalytic activities and synthesis process are discussed in the following sections as well as in Table 1. However, the photocatalytic performances of metal oxide-GO/rGO composite photocatalysts for the degradation of different pollutants are tabulated in Table 2.

### 2.1. Earth-Abundant Metal Oxide-GO/rGO Composites

#### 2.1.1. rGO-WO_3_ Composites

rGO-WO_3_ catalyst dosage has a substantial impression on the photocatalytic activity. The finest Sulfamethoxazole (SMX) removal efficacy was achieved when rGO-WO_3_-200 loading was 1.0 g L^−1^, and this study demonstrated that the dosage of the catalyst is crucial for the photocatalytic activity. SMX degradation over rGO-WO_3_-200 was significantly influenced by neutral pH and displayed the pre-eminent process [18]. Photodegradation of naphthol-1 by rGO-WO_3_–nanocomposite and WO_3_ was achieved by 84% and 40% respectively, attributed to the larger specific surface area and lower band gap energy [19]. Outcomes suggested that the presence of rGO in the nanocomposite enabled the electron transfer [19]. The enhanced photocatalytic activity was due to the synergistic effect between WO_3_ and rGO sheets and suppressing the electron-hole pair recombination in the WO_3_-rGO nanocomposite [6]. WO_3_/GO composites revealed an enhanced photocatalysis under the visible light, which was two-folds of pure WO_3_ that reduced the recombination of the photogenerated electron-hole pairs and increased the visible light absorption efficiency [20].

#### 2.1.2. rGO-Co_3_O_4_ Composites

Co_3_O_4_/rGO composite exhibits better photocatalytic activity in a low concentration of Methyl Orange (MO). The higher stocking dose of Co_3_O_4_ on Co_3_O_4_/rGO contributes more on the activity [21]. Degradation of organic dyes depicted by photocatalytic experiments where GO acts as a supporting material and active co-catalyst, which decreases the band gap of *α*-MoO_3_ from 2.82 to 2.51 eV [22]. This synthesized hybrid can be used for visible-light-induced photocatalysis. Co_3_O_4_-rGO hybrids were reported to completely oxidize 20 mg/L phenol in 20 min at 25 °C. Origination of sulfate radicals via Co_3_O_4_-mediated activation of peroxymonosulfate (PMS) is responsible for this catalytic effect [23]. The Co_3_O_4_/N-doped graphene hybrid exhibits similar catalytic activity but superior stability to Pt, in alkaline solutions. This unusual catalytic activity arises from synergetic chemical coupling effects between Co_3_O_4_ and graphene [23].

#### 2.1.3. GO/rGO-TiO_2_ Composites

**Figure 1:**
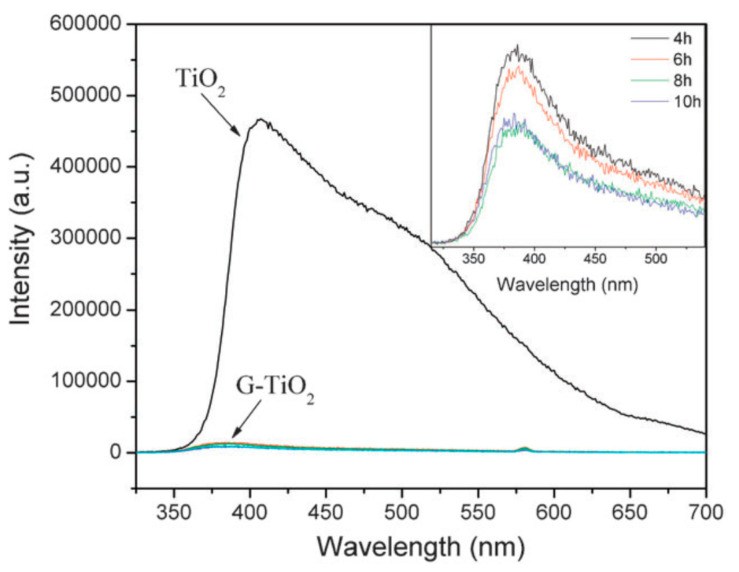
Photoluminescence spectra of TiO_2_ and G–TiO_2_ composite with various reaction times. The inset shows amplificatory image (300 to 550 nm) [26].

**Figure 2:**
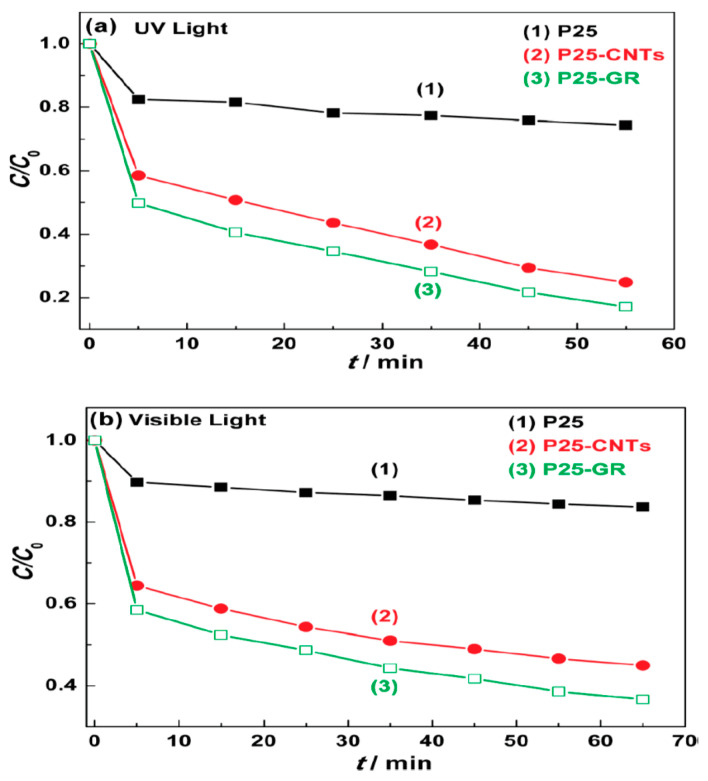
Degradation of Methylene Blue under (**a**) UV and (**b**) visible light (λ > 400 nm) over (1) P25, (2) P25-carbon nanotubes (CNTs), and (3) P25-GR photocatalysts, respectively [14].

A systematic investigation on the photocatalytic properties of TiO_2_–GO nanocomposites was examined with different ratios of graphene additions and it was found that higher amount of graphene addition decreased the photocatalytic activity [24]. Liquid-phase degradation of dyes over the TiO_2_-GO photocatalyst showed the similar occurrence. This study demonstrated that TiO_2_-GO cannot offer truly new visions into the assembly of TiO_2_ carbon composite as high-performance photocatalysts. The TiO_2_ particles were found to be in anatase phase and a narrow size distribution was dispersed on the surface of graphene sheets uniformly [25]. A comparison of photoluminescence spectra between TiO_2_ and G-TiO_2_ was reported with different reaction times, as shown in Figure 1. In this figure, the inset is the amplificatory image of the area in the range of 300 to 500 nm which demonstrates the quenching extent in relation with the reaction time in the Graphene-TiO_2_ [26]. TiO_2_ (P25)-rGO composite was found to be the most proficient photocatalyst for the degradation of Methylene Blue (MB) and the optimum mass ratio was found to be 1/0.2 [14]. Comparison has revealed that the P25-rGO composite has additional effectiveness compared to the P25-CNT (carbon nanotubes) composite (Figure 2).

Graphene-loaded TiO_2_ films were reported to be highly conductive and transparent; remarkably, graphene/TiO_2_ films exhibited super hydrophilicity in a short time even under a white fluorescent light bulb. Higher photocatalytic activity owed to its efficient charge separation and electrons injection from the conduction band of TiO_2_ to graphene [27].

The higher photocatalytic performance was observed in TiO_2_-graphene oxide composite due to the formation of both π-π conjugations between dye molecules and aromatic rings. The photocatalytic property was reported to be higher with the higher content of the graphene oxide. Furthermore, ionic interactions between MB and functional groups of GO on the surfaces of carbon-based nanosheets was also the reason considered for the superior property [28]. Improving graphene oxide (IGO) in strong acidic condition was reported to enhance the chemical interaction between TiO_2_ and graphene sheets [29]. This study showed that IGO can react with Ti(OH)_x_ to form graphene/TiO_2_ composite in situ, with complete and near coverage of Ti-C and Ti-O-C carbonaceous bonds at the graphene/TiO_2_ interface. Higher photocatalytic activity shown by graphene/TiO_2_ due to effective charge transfer imparts under visible light and GO forms chemical bonds at the interface [29].

Photocatalytic experiments using sacrificial hole and radical scavenging agents demonstrated that the photogenerated holes are the main reason for the degradation of diphenhydramine (DP), both under UV and visible light. In this report, photoluminescence studies revealed discrete appeasing of the GO photoluminiscence under visible light and near infrared laser excitation. Hence, it was conferred that GO acts as either an electron acceptor or donor of TiO_2_ under UV/visible light [30].

**Figure 3:**
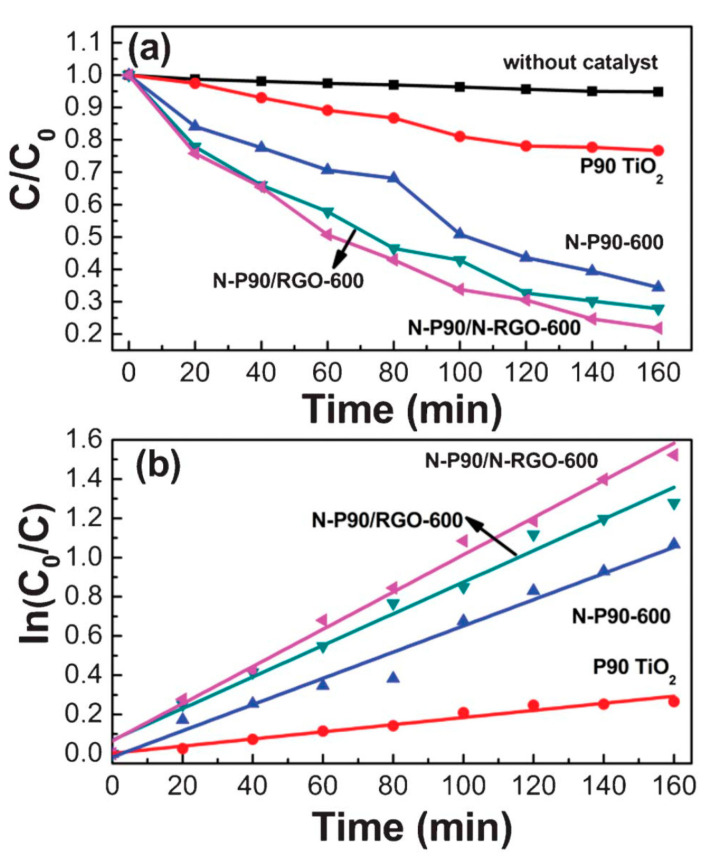
(**a**,**b**) Degradation of MB under visible light irradiation by P90 TiO_2_, N-P90-600, N-P90/reduced graphene oxide (rGO)-600, N-P90/N-rGO-600, and without catalyst [31].

Nitrogen-doped P90 TiO_2_ (N-P90), nitrogen-doped reduced graphene oxide (N-rGO), as well as their composites were studied for the photocatalytic activity. N-P90/N-rGO showed enhanced photocatalytic activity, and in the presence of this composite, around 80% MB was degraded by visible light irradiation in 160 min. Enhanced photocatalytic performance is observed in N-P90/N-rGO composites for degradation of MB due to photo-induced and electronic interaction between TiO_2_ and graphene. Comparison of degradation efficiency of MB under visible light irradiation by P90 TiO_2_, N-P90, and N-P90/rGO is presented in Figure 3 [31]. It is reported that the rGO or N-rGO in the composite enables the separation of electrons and holes by performing as electron trapping/de-trapping under visible light [31]. Ultrafine TiO_2_ nanofibers (~10 nm diameters) were synthesized from electrospun rice-shaped TiO_2_ and potassium titanate was achieved from rice-shaped TiO_2_. The surface area was increased by 2.5 times after the nanofiber formation of TiO_2_. The results showed that the photodegradation of MO was found to be higher than bare TiO_2_ (P-25) nanoparticles [32]. GO/TiO_2_ of different composition ratios were tested and the formulation of catalyst with 1.2 times higher photocatalytic activity than commercial photocatalyst was reported. This catalyst was able to degrade 3 mg/L MB over 10 consecutive cycles with nominal loss in photocatalytic efficacy. Graphene plays a generous part in obstructing the accretion of TiO_2_ grains upon calcination at high temperature [33]. A set of reduced graphene oxide-TiO_2_ (rGO-TiO_2_) nanocomposites was synthesized and examined for the photocatalytic activity by decolorization of Rhodamine B dye (RhB) under UV light. In this study, various parameters, such as dye concentration, rGO content, catalytic dose, and pH, were optimized for the decolorization. The catalysts were found to be more active at natural pH of the dye under the UV-illumination for the degradation of RhB dye. The presence of H_2_O_2_ and K_2_S_2_O_8_ increased the decolorization. Further, addition of CO_3_^2−^ and Cl^−^ ions decreased the dye degradation rate [34].

TiO_2_ nanoparticle-attached graphene/carbon composite nanofibers (TiO_2_-CCNFs) were synthesized and reported as highly active photocatalysts for photocatalytic degradation of MB under the irradiation of visible light. Graphene was suggested to play the role of an electron acceptor and a photosensitizer, resulting in a higher photodegradation rate and reduced electron-hole pair recombination. CNFs having high surface also improved the photocatalytic activity of TiO_2_ [35].

#### 2.1.4. GO/rGO-ZnO Composite

An effective scalable method was developed to make nanocomposites of functional graphene sheets (FGS)/ZnO. In this study, poly (vinyl pyrrolidone) (PVP) component was reported to play a crucial role for loading of ZnO nanoparticles onto FGS by connecting Zn ions on the carbon materials and promoting ZnO nucleation and crystal growth in the precursor-prepared route. Further, FGS/ZnO composite was evaluated for photocatalytic activity and was found to be applicable for a number of environmental issues [36]. ZnO/rGO nanocomposite was used as a photocatalyst for the removal of MB. Observations showed that the efficiency of the photocatalyst activity of the ZnO nanoparticles was significantly increased by rGO [37].

The calcination atmosphere was found to affect the photocatalytic activity of the TiO_2_/graphene sheet (GS) (5%) composites for H_2_ evolution from water splitting. This study demonstrated that beyond the critical content of GS (5%), photocatalytic activity was decreased by initiating electron-hole recombination centers. Calcination atmosphere was found to be important and better performance was observed for the samples calcined in nitrogen atmosphere [38].

**Figure 4:**
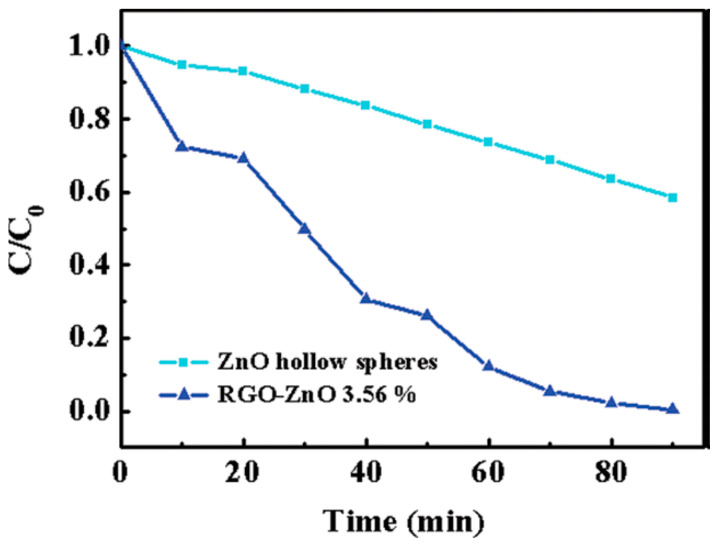
Photocatalytic activity of ZnO hollow spheres and rGO-ZnO 3.56% for the degradation of MB under UV illumination [40].

The use of ZnO-graphene composites (Z-GC) was reported to remove dye from water due to the interaction between the graphene sheets and the ZnO nanoparticles [39]. rGO-ZnO (3.56%) showed higher photocurrent response and degradation of MB under illumination of UV light. Longer electron lifetime and the enhanced light absorption were verified by analytical and electrochemical technique (Figure 4) [40].

The ZnO/GO nanocomposite consisting of flower-like ZnO nanoparticles anchored on graphene-oxide. Further, photocatalytic efficiency of ZnO/GO composite progressed by annealing the product in N_2_ atmosphere. The superior photocatalytic performance was due to the synergistic effect of the proficient electron inoculation and low charge carriers in the composite, where GO acted as an electron collector and transporter, leading to unceasing generation of reactive oxygen species for the degradation of MB [10]. Core-shell nanorods with ZnO core and ZnS-Bi_2_S_3_ bi-component shell anchored on the rGO sheets were synthesized and reported to show a broad and strong photo-absorption in the visible region. These nanorods also manifested better photocatalytic activity for H_2_ evolution from the glycerol-water mixtures. The superiority in performance is owing to the elevated light absorption and effective charge separation [41].

### 2.2. Bimetal Oxide-GO/rGO Composites

#### 2.2.1. GO/rGO-CoFe_2_O_4_ Composite

Connexion of the graphene suggestively progressed the photocatalytic performance of the CoFe_2_O_4_ in which the graphene acts as a charge carrier to detain the delocalized electrons. Photocatalytic activity was explored with the variation of the dosage and dye concentration [42]. CoFe_2_O_4_-graphene hybrid materials (CFGHs) showed ferromagnetic behavior and enhanced photodegradation rate and amended adsorbing capacity due to the assimilation of graphene [43]. The photodegradation fallouts directed the visible light fascinating performance of the ternary photocatalysts and formation of the p–n junction between CoFe_2_O_4_ and CdS. Escalation in the concentration of MB was observed as the irradiation time increased for CoFe_2_O_4_ due to the desorption of MB during irradiation. G-CoFe_2_O_4_/CdS easily separated from aqueous solution in an external magnetic field, as seen from the digital photos of Gr-CoFe_2_O_4_/CdS after irradiation [44]. The CoFe_2_O_4_-rGO composite unveiled required photocatalytic performance with excellent recycling stability for the degradation of MB, RhB, and MO under visible-light irradiation [45].

CoFe_2_O_4_-rGO (CF–rGO) nanocomposites hold exceptional microwave absorbing properties and high photocatalytic activity for the degradation of various dyes under visible light irradiation [46]. 85 CF-15 rGO exposed admirable microwave absorption possessions with a Reflection Loss (RL) of 31.31 dB (99.94% absorption) at 9.05 GHz, with an 8.2–10.92 GHz effective bandwidth range. 75CF-25 rGO was found to be a good magnetically separable photocatalyst for the degradation of dyes, MO, MB, and RhB, under visible light irradiation emitted from a 100 W reading lamp [46]. The photocatalytic activity was found to be affected by the structural and optical properties and surface area of the samples [47].

CoFe_2_O_4_-3D TiO_2_ nanocomposite showed an enhancement in the photodegradation of MB as compared to the commercial rutile-phase TiO_2_ and the pure urchin-like TiO_2_ (3D TiO_2_) microparticles. Results specified that the composite showed relatively consistent photocatalytic activity with slight degradation [48]. The photocatalytic activity of 75CF-25 rGO was found to be analogous and in some cases, superior, compared to the several reported rGO–CoFe_2_O_4_ composites [46]. The photocatalytic activity of CF-RGO was increased with increasing rGO content in composites until 25 wt% of rGO, and degradation takes place around 60 min.

**Figure 5:**
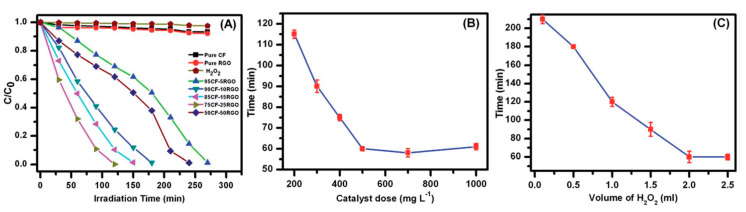
(**A**) Different catalysts for the degradation of Methyl Orange (MO) under visible light. (**B**) Catalyst dosage and (**C**) H_2_O_2_ on the accomplishment time of photocatalysis reaction of MO catalyzed by 75CF-25RGO [46].

**Figure 6:**
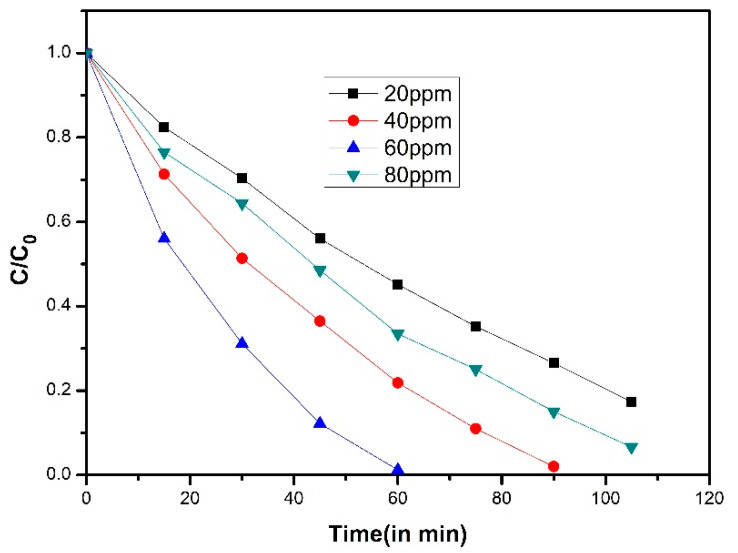
Plot of C/C_o_ vs. time in min for the degradation of 4-CP (10 ppm) with 100 mg of catalyst at various peroxymonosulfate (PMS) concentrations [50].

The photocatalytic degradation of short-chain chlorinated paraffin’s over rGO/CoFe_2_O_4_/Ag under visible light (λ > 400 nm) was investigated by in-situ Fourier transform infrared spectroscopy and the correlated mechanisms were suggested. Superficial degradation ratio of 91.9% over rGO/CoFe_2_O_4_/Ag was obtained under visible light illumination of 12 h, while only about 21.7% was obtained with commercial P-25 TiO_2_ [49]. Increase of rGO caused an increase in the completion time of the photocatalysis. Degradation of MO diminished with increasing catalyst dose up to 500 mgL^−1^, and then, no noteworthy decrease of time was observed when more catalyst was added. Likewise, use of 2 mL of H_2_O_2_ was found to be an optimum amount for the photocatalysis reaction (Figure 5) [46]. Photocatalytic activity of the rGO-CoFe_2_O_4_ nanocomposites was queried for the degradation of 4-Chlorophenol (4-CP) under visible light illumination. Activity of rGO-CoFe_2_O_4_ composite was seen in the occurrence of PMS (Figure 6) [50].

#### 2.2.2. GO-rGO-ZnFe_2_O_4_ Composite

Photocatalytic activity of ZnFe_2_O_4_-graphene catalyst demonstrated an important two-fold function as the photoelectrochemical degradation of MB and generation of hydroxyl radical for the decomposition of H_2_O_2_ under visible light irradiation [51]. Graphene-ZnFe_2_O_4_ photocatalyst facilitated the transport channels for photon-excited electrons from the surface of the catalyst. As a result, about 20 nm ZnFe_2_O_4_ catalyst with a highly crystallized (311) plane confined in the graphene network exhibited an excellent visible-light-driven photocatalytic activity with an ultrafast degradation rate of 1.924 × 10^−7^ mol g ^−1^ s ^−1^ for MB [52].

The boosted photocatalytic activity of ZnFe_2_O_4_-rGO nanocomposite was shown due to the active restraint of the recombination of the photo-excited electron-hole pairs by rGO sheets and the generation of ·OH-free radical [11]. The photocatalytic activity of the nanocomposite examined under visible light, for the degradation of 17 α-ethinylestradiol (EE_2_) [50]. The pseudo rate constant of ZnFe_2_O_4_-Ag/rGO nanocomposite was higher by the factor of 14.6 and 5.6 times over its counterparts. Photosensitization effect was prevailed by good interaction ensuing in only 80% removal of EE_2_ though humic acid [53]. rGO/ZnFe_2_O_4_ composite exhibited the remarkable catalytic activity toward MB degradation; in the presence of H_2_O_2_, the activity enhanced, and the reaction followed a pseudo-first-order kinetics. The complete MB degradation observed at rGO/ZnFe_2_O_4_ composites was attributed to the π–π interaction, hydrogen bonding, and electrostatic interaction exerted between the rGO and ZnFe_2_O_4_ [54].

#### 2.2.3. GO/rGO-NiFe_2_O_4_ and MnFe_2_O_4_ Composites

**Figure 7:**
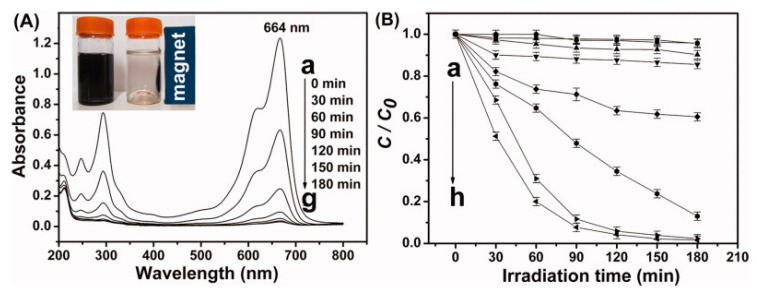
Absorption spectra of MB taken at various photocatalytic degradation times consuming NiFe_2_O_4_-GO (0.25) [55]. (**A**) The magnetic separation property of NiFe_2_O_4_-graphene nanocomposite is shown in the inset, (**B**) different catalysts and their photocatalytic degradation efficiency on MB: (**a**) pure NiFe_2_O_4_, (**b**) NiFe_2_O_4_-G(0.05), (**c**) NiFe_2_O_4_-G(0.10), (**d**) NiFe_2_O_4_-G(0.15), (**e**) NiFe_2_O_4_-G(0.20), (**f**) NiFe_2_O_4_-G(0.40), (**g**) NiFe_2_O_4_-G(0.30), and (**h**) NiFe_2_O_4_-G(0.25).

NiFe_2_O_4_-GO (0.25) hetero-architecture demonstrated a considerable lesser emission intensity. Due to their competent electron-transport property, graphene sheets can deliberately reduce the fluorescence of NiFe_2_O_4_ fixed on them. Kinetic results indicated that the rate-determining step is the adsorption course of MB [55]. In this study, NiFe_2_O_4_-GO (0.25) shows the best activity compared to other NiFe_2_O_4_-G composites (Figure 7). GO-NiFe_2_O_4_ showed photo-Fenton reactions for organic contaminants in the presence of both H_2_C_2_O_4_ and H_2_O_2_ under visible light irradiation. The photochemical reduction of Fe^3+^ ions by GO was a key step in inducing the Fenton process [56]. The superior photocatalytic is due to (I) high visible absorbance for charge carrier production, (II) the electrons captured by Au nanoparticles results in the fast separation, and (III) the strong surface plasmon resonance (SPR) of Au nanoparticles permit the generation of high concentration of charge carriers [57]. MnFe_2_O_4_ catalyst is photocatalytically inactive. The noteworthy higher photocatalytic activity is due to the rGO, as the excellent conductivity in the MnFe_2_O_4_ and graphene composite [58].

#### 2.2.4. Other Composite Systems

RGO-Bi_2_WO_6_ and 3D CNT-pillared rGO nanocomposites show outstanding photocatalytic performance for the degradation of dyes under visible light [59]. BiFeO_3_-graphene nanohybrids have a six times higher rate compared to BiFeO_3_ for the degradation of Congo Red (CR) under visible light due to its combined effects of modulated band gap and covalent bonding between BiFeO_3_ and graphene [60].

Photoluminescence studies of Nb_3_O_7_ (OH)-RGO composite supported the suggested mechanism of charge separation and transport mechanism. A higher degradation rate was obtained using the nanocomposite prepared with a graphene loading of 3 mgmL^−1^, and when the rGO loading exceeded 3 mgmL^−1^, degradation efficacy diminished. This arose as extra rGO sheets gathered and stuck the absorption of incident light [61].

## 3. Photocatalytic Evaluation

Pristine TiO_2_ and ZnO exhibited good photocatalytic activity in UV light due to their wide band gap. These two metal oxides are stable in aqueous conditions during photocatalysis. Further, coupling of graphene with TiO_2_ and ZnO increases the photocatalytic activity due to increases in the photogenerated charge carriers. Metal oxides with magnetic properties of metal ferrites (MFe_2_O_4_) offer an added advantage as photocatalysts since they can be recovered by applying an external magnetic field after catalysis. Metal ferrites (MFe_2_O_4_, M = Co, Ni, Mn, Zn, etc.) materials are proven to be excellent candidates for visible light photocatalytic H_2_ generation through water splitting. Recycling ability for metal ferrites are far better compared to nano semiconductors like TiO_2_ and ZnO. MFe_2_O_4_ is a class of semiconductor with narrow band gap, which exhibits characteristic visible light response, possess good photochemical stability, and exhibits excellent optical properties.

MFe_2_O_4_ absorbs 42–45% of sunlight, whereas TiO_2_ and ZnO absorbs 4% of sunlight. MFe_2_O_4_ are efficient for the degradation of dye degradation and organic pollutant degradation compared to the other metal oxides (SnO_2_, CeO_2_, BaTiO_3_, and SrTiO_3_), with respect to the catalyst and the light source. In MFe_2_O_4_ context, recombination of photogenerated charge carriers is the major limitation in semiconductor photocatalysis as it reduces the overall quantum efficiency. In order to enhance the photocatalytic activity, graphene material is coupled with MFe_2_O_4_, where the graphene channels the electrons. Comparison of degradation rate for various photocatalytic reaction systems is incongruous since the nature of catalyst and substrate pollutant molecules are different in each reaction. Ferrite nanoparticles have a strong magnetic property, which can be easily used for magnetic separation after photo-mineralization.

The photocatalytic efficiency depends on the ratio of the photogenerated charge-carrier transfer rate to the rate of electron-hole recombination. For composite structure, M^2+^ ion easily bonds with oxygen by giving an electron and super oxide radical. This super oxide radical can oxidize the organic substrate molecule. The Fe^3+^ ion and Fe^2+^ ions can show photo-Fenton reactions in presence of in-situ-generated H_2_O_2_. This H_2_O_2_ generates hydroxyl-free radicals, which are involved in the degradation of pollutants. Predicted mechanism for the rGO-CoFe_2_O_4_ composite is shown in Equations (1)–(4).

(1) $$\mathrm{rGO}(e^{-})-{\mathrm{CoFe}}_{2}O_{4}(h^{+})+O_{2}\to \mathrm{rGO}-{\mathrm{CoFe}}_{2}O_{4}(h^{+})+O_{2}^{•-}$$

(2) $$\mathrm{rGO}-{\mathrm{CoFe}}_{2}O_{4}(h^{+})+{\mathrm{OH}}^{\_}/H_{2}O\to \mathrm{rGO}-{\mathrm{CoFe}}_{2}O_{4}{+}^{•}\mathrm{OH}$$

(3) $$\mathrm{rGO}(e^{-})-{\mathrm{CoFe}}_{2}O_{4}(h^{+})+{\mathrm{HSO}}_{5}^{-}\to \mathrm{rGO}-{\mathrm{CoFe}}_{2}O_{4}{+}^{•}\mathrm{OH}+{\mathrm{SO}}_{4}^{•-}$$

(4) $${\mathrm{SO}}_{4}^{•-}+H_{2}O\to {\mathrm{HSO}}_{4}^{-}{+}^{•}\mathrm{OH}$$

rGO-BiO_6_ composite shows better photocatalyst compared to other catalysts prepared from hydrothermal method (Table 1; Table 2). The enhanced photocatalytic activity could be endorsed to the negative shift in the Fermi level of graphene-Bi2WO6 (G-BWO), decrease the conduction band potential, and elevate migration efficiency of photo-induced electrons, which may restrain the charge recombination efficiently. Superior contact between BiVO_4_ and rGO scaffold subsidizes to photo-response augmentation compared to other electrochemical methods in the rGO-BiO_4_ composite.

Furthermore, the self-redox properties of iron and manganese atoms in MnFe_2_O_4_ induced by S_2_O_8_^2−^ were particularly useful for the generation of SO_4_^−^. The quenching tests and electron spin resonance (ESR) display that *h*^+^, O^2−^, SO_4_^−^, and OH are accountable for decomposition of antibiotics. Overall, irrespective of other parameters, the solvothermal method is best and helps in crystal growing and super saturation is achieved by reducing the temperature in the crystal growth zone.

Further, noble metal (Ag, Au, Cu, etc.) exhibits surface plasmon resonance (SPR), which is a characteristic feature. The SPR frequency of the metal particles can be tuned into visible light absorption by shifting the size of the deposited metal particles on the catalyst. Deposited metal is involved in multiple crucial roles, such as serving as a passive electron sink with high capacity to store electrons to suppress photogenerated charge carrier recombination, facilitates rapid dioxygen reduction to generate free radicals and direct excitation of metals, especially under visible light, and vectorial electron transfer to the conduction band (CB) of metal oxide. Thereby, showing improvement in the photocatalysis for the removal of various organic pollutants/dyes.

## 4. Perspectives and Challenges

Graphene nanosheets act as a substrate to support the metal oxides for photocatalytic activity and graphene-based semiconductor photocatalysts are used for environmental remediation. The morphologies of semiconductors, theoretical electronic-structure calculations, and experimental discovery determinations are necessary on GO to persuade the photocatalytic activity, and composition design is an operative method to enhance the photocatalytic properties. Photocatalytic properties depend on the preparative method, and various parameters like initial concentration, oxidant concentration, pH, particle size, number of GO sheets, and source of light should be explored.

The interface regulates the efficacy of the electron-hole separation. Currently, only few methods succeed in unswervingly depicting the interaction of GR and nanoparticles. Atomic force microscopy (AFM), Surface-enhanced Raman scattering (SERS), and scanning transmission electron microscope (STEM) may be the best techniques for determining the interaction of graphene and nanoparticles. Finally, studies on the preparation of a ternary composite as a photocatalyst for both UV and visible-light-driven pollutant photodegradation have been studied and reported. Especially, for the design of ternary composite, magnetic materials such as Fe, Co, Mn, etc., as a dopant, and possessing unique advantages, show a remarkable photocatalytic activity and photostability.

Further tasks exist in the application of graphene-based composite for the industrial scale. Some innovative applications of the metal oxide-graphene entail specific understanding between the metal oxides and surface of the graphene, which will have a direct impact on the properties of the composite. Designing a structure for the overall photocatalysis process may require further exploiting of GO by chemically modifying methods. A synthetic approach method of GO-based composite structure by using novel materials has not been achieved to date for photocatalysis, but the solutions to the key challenges appear within reach.

In view of this, graphene-based composites possess diverse potential applications, individually having dissimilar desires concerning material properties, and it can be projected that the research on graphene-composite materials will have an optimistic future.

## Figures and Tables

**Table 1:** Photocatalysts and their typical synthetic methods used for the preparation from GO/rGO-supported composites.

Order	Photocatalyst	Preparation	References
**A.** **Hydrothermal method for the synthesis of GO/rGO-NCs photocatalyst**			
1	rGO-WO_3_	Na_2_WO_4_·2H_2_O and 0.05 g NaCl were dissolved in the above dispersion and kept stirring for 1 h. The pH was adjusted to 2 by using HCl solution.	[6]
2	rGO-WO_3_	Preset amounts of Na_2_WO_4_·2H_2_O (100, 200, and 400 mg, respectively) were dissolved in 10 mL above GO suspension. 5 mL 35% HCl was added slowly. Transferred to autoclave heated at 140 °C for 8 h.	[18]
3	rGO_Co_3_O_4_	GO dispersed into 24 mL of alcohol, sonicating for 60 min in an ultrasonic cleaner. Then, 0.2 M of Co(Ac)_2_ was added to the mixture followed by 1.2 mL of water, and continued to be stirred for 10 h at a temperature of 80 °C. The resulting solution was then transferred into a 40 mL autoclave for hydrothermal reaction at 150 °C for 3 h.	[21]
4	rGO_Co_3_O_4_	GO dispersed in the Co (C_2_H_3_O_2_)_2_·4H_2_O. 10 mL with 28% ammonia solution were added to solution, and transferred into an autoclave for hydrothermal action at 180 °C for 12 h.	[23]
5	rGO/ZnFe_2_O_4_-Ag	The composite was synthesized by the co-precipitation of Zn (NO_3_)_2_·6H_2_O, Fe (NO_3_)_3_.9H_2_O, and AgNO_3_ in the presence of the GO powder.	[53]
6	GO-NiFe_2_O_4_	GO in NiFe_2_O_4_ was dispersed in deionized water. Then, NiSO_4_H_2_O and FeCl_3_6H_2_O (0.02 mol) were dissolved in 15 mL water. Transferred into autoclave and kept under high pressure.	[56]
7	GO-MnFe_2_O_4_	GO and 60 mL of ethanol with sonication for 1 h and Mn(NO_3_)_2_ solution and Fe (NO_3_)_3_ 9H_2_O were dissolved. The resulting mixture was transferred into a 100 mL Teflon-lined stainless-steel autoclave and heated to 180 °C for 20 h under autogenous pressure.	[58]
8	rGO-Bi_2_WO_6_	GO by using Hammer method GO was reduced by ethylene glycol. Bi(NO_3_)_3_ 5H_2_O was dispersed into 5 mL of 4 M nitric acid solution. Na_2_WO_4_ 2H_2_O was dissolved in 5 mL of de-ionized water and then Na_2_WO_4_ was added dropwise to the solution.	[59]
**B.** **Sol-gel method for the synthesis of GO/rGO-NCs photocatalysts**			
9	rGO-TiO_2_	An aqueous solution of Ti(OH)_4_ was added into an aqueous suspension of GO.	[30]
10	rGO-ZnO	An aqueous solution of Zn (AcO)_2_·3H_2_O was added into an aqueous suspension of GO.	[39]
**C.** **Solvothermal technique for the GO/rGO-NCs photocatalysts**			
11	GO/CoFe_2_O_4_/CdS	Gr–CoFe_2_O_4_ nanohybrids were sonicated in 60 mL of ethylene glycol for 10 min. The cadmium source containing 0.1431 g of Cd(NO_3_)_2_4H_2_O and10 mL of ethylene glycol was added to the mixture containing Gr–CoFe_2_O_4_ nanohybrids. The mixture of 0.0348 g of thiourea, 0.0514 g of PVP, and 10 mL of ethyleneglycol was transferred into the above mixture.	[44]
12	rGO-ZnFe_2_O_4_	GO dispersed in ZnO_x_(OH)_y_ and FeO_x_ solutions were put into a 50 mL autoclave.	[51]
**D.** **Colloidal method for the synthesis of GO/rGO-NCs photocatalyst**			
13	rGO-ZnFe_2_O_4_	C_2_H_6_O_2_ solution is containing 2M FeCl_3_6H_2_O, and 1M ZnCl_2_ was gradually added. Then, 1MCH_3_COONa was introduced into the solution and magnetically stirred for 1 h. Then, transferred to autoclave heated at 1800 °C.	[54]
**E.** **Thermal treatment for the synthesis of GO/rGO-NCs photocatalyst**			
14	rGO-WO_3_	Na_2_WO_4_·2H_2_O was dissolved in 30 mL water. Then, nitric acid was added to the solution drop by drop until the precipitate was formed. Dried at 160 °C for 2 h and annealed at 500 °C for 5 h.	[19]
15	rGO-WO_3_	Na_2_WO_4_·2H_2_O (0.5 g), H_2_C_2_O_4_ (1 g), and Na_2_SO_4_ (4 g) were added into subsequent solution and stirred for 3 h. The pH of the solution was maintained at 1.5 by adding 3M HCl and stirring was continued for 3 h. Then, transferred to autoclave maintained at 180 °C for 24 h.	[20]
16	FGS/ZnO	GO, Zn(NH_3_)_4_CO_3_, and poly(vinyl pyrrolidone) as an intermediate to combine zinc with carbon material	[36]
**F.** **Ball-milling method for the synthesis of GO/rGO-NCs photocatalyst**			
17	rGO-CoFe_2_O_4_	Co (NO_3_)_2_·6H_2_O and of Fe (NO_3_)_3_·9H_2_O were added to GO (2.5 wt%). The pH is maintained 10	[45]
18	rGO-CoFe_2_O_4_		[45]
19	rGO-CoFe_2_O_4_		[45]
**G.** **Liquid phase deposition method for the synthesis of GO/rGO-NCs photocatalyst**			
20	rGO-TiO_2_	TiO_2_ powder (P25, Degussa) was dispersed in deionized water and subsequently added to the graphene oxide solution	[28]
**H.** **Microwave irradiation method for the synthesis of GO/rGO-NCs photocatalyst**			
21	rGO-CoFe_2_O_4_	(Co(NO_3_)_2_·6H_2_O and Fe(NO_3_)_3_ 9H_2_O and glucose as oxidizer and fuel. GO, nitrates, and glucose were added in water for 30 min ultrasonic treatment.	[43]
22	rGO-CoFe_2_O_4_		[43]
23	rGO-CoFe_2_O_4_		[43]
24	rGO/CoFe_2_O_4_/Ag	GO, AgNO_3_, and CoFe_2_O_4_ were dissolved in deionized water and stirred for 2 h. Then, solution was further stirred for 2 h under the UV irradiation of a 22 W low-pressure mercury lamp. The product is washed with distilled water and ethanol in an oven at 60 °C for 12 h.	[49]
**I.** **In situ co-precipitation method for the synthesis of GO/rGO-NCs photocatalyst**			
25	rGO-ZnO	GO dispersed in aqueous solution containing Zn(CH_3_COO)_2_, DMSO, and H_2_O	[40]
**J.** **Annealing NH_3_ atmosphere method for the synthesis of GO/rGO-NCs photocatalyst**			
26	rGO/N-TiO_2_	GO and 300 mg of P90 TiO_2_ was added and stirred for 3 h. GO and P90 TiO_2_ and a few drops of tetrabutyltitanate were added.	[31]

**Table 2:** Photocatalytic performances of GO/rGO-NCs photocatalysts for the degradation of pollutant.

Order	Pollutants	Photocatalyst	Light Source	Reactor	Mass of Catalyst (mg)	Concentration (ppm)	Irradiation Time (min)	Conversion (%)	Mol. Wt.	Photon Flux (mW cm^−2^)	Quantum Yield (%)	Reference
**A.** **Photocatalytic performances of GO-rGO semiconductor composites for dye degradation**												
**A-1. MB**												
1	MB	rGO-WO_3_	light source was a 150 W xenon lamp.	20 °C self-made Lab Solar gas photocatalysis system with external light irradiation	50	7	120	100	2278.4	NA	NA	[6]
2	MB	rGO-WO_3_	One 300 W PLS-SXE 300 xenon lamp	equipped with a λ < 400 nm cut-off filter	20	10	70	95	2247.4	NA	NA	[20]
3	MB	rGO/N-TiO_2_	Two 20 W black-lights with 352 nm (UV) and 545 nm (Visible)	NA	10	8.8	60	80 (UV) and 95 (Visible)	2123.6	NA	NA	[28]
4	MB	rGO/N-TiO_2_	one 500 W Xenon lamp > 400 nm	Quartz cell	50	8.8	160	100	2137.6	NA	NA	[31]
5	MB	rGO-ZnO	one 300 W Xe lamp with 420 nm	NA	80	18	70	100	2125.2	NA	NA	[39]
6	MB	rGO-ZnO	one 500 W mercury lamp	NA	20	10	90	100	2125.2	NA	NA	[40]
7	MB	GO/CoFe_2_O_4_/CdS	one 40 W daylight lamp	NA	25	20	180	100	2422.8	NA	NA	[44]
8	MB	rGO-CoFe_2_O_4_	one 800 W Xe lamp	NA	10	20	180	100	2278.4	NA	NA	[45]
9	MB	rGO-CoFe_2_O_4_	A 100 W reading lamp	installed glass cut-off filter	25	10	75	90	2278.4	NA	NA	[46]
10	MB	rGO-ZnFe_2_O_4_	one 500 W xenon lamp > 420 nm	Glass reactor (100 mL)	50	10	90	61	2284.7	NA	NA	[51]
11	MB	rGO-ZnFe_2_O_4_	one 530 W lamp with >400 nm	Pyrex glass tube (100 mL)	25	10	120	100	2284.7	NA	NA	[54]
12	MB	NiFe_2_O_4_-GO	One 300 W UV-visible lamp	Quartz glass (100 mL)	100	20	600	90	2280.2	NA	NA	[56]
13	MB	MnFe_2_O_4_-GO	one 500 W mercury and xenon lamp	Glass tube (100 mL)	25	20	360	98	2274	NA	NA	[58]
**A-2. MO**												
14	MO	rGO_Co_3_O_4_	One 100 W Xenon lamp	NA	10	30	180	80	2287	NA	NA	[21]
15	MO	rGO-TiO_2_	one 150 W medium-pressure mercury vapor lamp with >350 nm	quartz cylindrical reactor (7.5 mL)	100	500	30	100	2123.6	6	NA	[30]
16	MO	rGO-CoFe2O4	one 800 W Xe lamp	NA	10	20	180	25	2278.4	NA	NA	[45]
17	MO	rGO-CoFe2O4	A 100 W reading lamp	installed glass cut-off filter	25	10	75	60	2278.4	NA	NA	[46]
**A-3. RhB**												
18	RhB 6G	FGS/ZnO	Two 100 and 250 W high-pressure mercury lamps with 300 nm	Pyrex glass tube (1000 mL)	10	10	100	100	2125.2	NA	NA	[36]
19	RhB	rGO-CoFe_2_O_4_	one 800 W Xe lamp	NA	10	20	180	75	2278.4	NA	NA	[45]
20	RhB	rGO-CoFe_2_O_4_	A 100 W reading lamp	installed glass cut-off filter	25	10	75	90	2278.4	NA	NA	[46]
21	RhB	rGO-Bi_2_WO_6_	one 500 W Xe lamp with 420 nm	Installed glass cut-off filter to visible-light irradiation glass tube (500 mL)	100	0.355	15	98	2740.8	NA	NA	[59]
**B.** **Photocatalytic performances of GO-rGO semiconductor composites for organic pollutants degradation**												
22	sulfamethoxazole	rGO-WO_3_	200 W Xe arc lamp with specific ranges 420–630 nm	1.5 AM solar simulator	10	20	180	100	2247	NA	NA	[18]
23	1-Naphthol	rGO-WO_3_	One Xe lamp 570 W	cylindrical Pyrex reactor of 7 cm diameter and 15 cm height	50	150	120	100	2247	NA	NA	[19]
24	Chain chlorinated paraffin’s	RGO/CoFe_2_O_4_/Ag	One 500 W xenon lamp with 400 nm	in situ quartz reaction cell	10	NA	720	90	2386.2	NA	NA	[49]
25	17 α-ethinylestradiol	rGO/ZnFe_2_O_4_-Ag	One 300 W Xe-lamp	NA	100	2	240	100	2382	NA	NA	[53]
